# Health-related quality of life of Palestinian preschoolers in the Gaza Strip: a cross-sectional study

**DOI:** 10.1186/1471-2458-11-253

**Published:** 2011-04-21

**Authors:** Salwa G Massad, F Javier Nieto, Mari Palta, Maureen Smith, Roseanne Clark, Abdel-Aziz Thabet

**Affiliations:** 1 Department of Nutrition and Dietetics, BirZeit University, BirZeit, Palestinian Territory; 2 Department of Population Health Sciences, University of Wisconsin-Madison; Madison, USA; 3 Department of Psychology, University of Wisconsin-Madison; Madison, USA; 4 Child Institute, Al Quds University - Gaza Branch, Gaza, Palestinian Territory

## Abstract

**Background:**

Research on children's responses to wartime trauma has mostly addressed Post-Traumatic Stress Disorder (PTSD). However, PTSD is only one aspect of a complex set of responses. This study proposes to expand knowledge of well-being in children exposed to political violence through widening the conceptualization of well-being beyond PTSD, morbidity, and mortality by measuring health-related quality of life (HRQOL) and its facets, physical health, and psychosocial health.

**Methods:**

In 2007, we used a cross-sectional random sample of kindergartens to examine factors associated with HRQOL, as measured by the PedsQL 4.0, in 350 preschoolers in the Gaza Strip, Palestine, where political violence and deprivation are widespread.

**Results:**

About 65% of the mothers reported severely impaired psychosocial and emotional functioning in their children. Preschoolers had lower HRQOL than the US reference sample and samples of children in other low income countries with large effect size. HRQOL was comparable to those of US children with several chronic diseases. Factors associated with lower HRQOL were older child age, male gender, and more exposures to traumatic events. Factors associated with HRQOL subscales were for lower psychosocial health: older child age, history of food, water, and electricity deprivation during incursion, and witnessing assassination of people by rockets. For lower physical health: older child age, history of food, water, and electricity deprivation during incursion, and having heard of a killing of a friend by soldiers.

**Conclusions:**

HRQOL, including psychosocial health and emotional functioning is often severely impaired among preschoolers in the Gaza Strip. Exposure to both violent and non-violent negative events was associated with HRQOL in preschoolers.

## Background

Direct and indirect exposure to violence is common among children in Palestine, and especially among those living in the Gaza Strip [1-3]. Political violence has resulted in the imposition of restrictions on the movement of Palestinian goods and people across borders and within Palestine. These restrictions have seriously compromised household welfare, resulting in loss of income, decreased quantity and quality of food, and impeded access to health care [4]. Since this time, poverty has risen dramatically, with more than 57% of the Palestinians (47% in the West Bank, 77% in the Gaza Strip) currently living below the poverty line (less than $2/person/day) [5].

Current knowledge about children's responses to wartime trauma is limited mostly to Post Traumatic Stress Disorder (PTSD) [6]. PTSD is defined as an anxiety disorder developed as a result of exposure to a terrifying event or ordeal [6]. However, PTSD is only one extreme aspect of a complex set of responses [6]. Health-Related Quality of Life (HRQOL) is another important measure of the health costs of war. We are not aware of studies measuring HRQOL among children in conflict areas. Most studies of HRQOL were among children with chronic diseases [7-11]. Traditional measures of morbidity and mortality may be too narrow in focus to capture the range of health issues in children [12], especially among those in situations of severe adversity. Child health outcomes also include broader health concerns, such as physical, emotional, social, and school functioning [12], which is measured in health-related quality of life. Availability of data on HRQOL in children will not only help identify subgroups at risk, but may also assist in the evaluation of health care needs and in the allocation of resources [12].

Research on the effects of war on children found that exposure to war leads to severe stress reactions and anxiety in a significant number of children [13]. Child witnesses of violence show an increased incidence of internalizing behaviors, such as depression, and externalizing behaviors, such as aggressiveness and noncompliance [14]. In 2001, a national survey of the impact of political violence on the well-being of Palestinian children age 5-17 years found that 44% had crying attacks, 41% had fear of blood color, 28% thought too much about death, and 22% had anger and nervous breakdown [2]. In 2003, Qouta investigated the prevalence of mental health problems of 121 Palestinian children (aged 6-16 years) and found that 54% suffered from severe levels of PTSD [15]. Similarly, in a study of Kuwaiti children following the Persian Gulf War, more than 70% of the children reported moderate or severe PTSD [16]. The reactions to being exposed to violence intensify with increasing level and duration of violence [14,17-19]. In 2003, Thabet examined the behavioral and emotional problems of 309 Palestinian preschoolers and found that direct and indirect exposure to war trauma increases the risk of poor mental health [20].

Most previous research examined the impact of exposure to violence as the only stressor affecting the mental health of children in war [6,20-24]. In addition to traumatic events, however, it is equally important to examine secondary stressors such as deprivation [25] and forced relocation [26,27]. Moreover, as we have demonstrated elsewhere [28], studying predictors of resilience may offer complementary insights. Parental social support, for example, predicts resilience in poor families [29]; social support may improve access to cash loans and food, esteem, status, motivation, information, companionship, emotional empathy, and understanding [30,31]. Far less studied has been the influence of maternal mental health on child mental health [32]. The few studies that have examined the effect of maternal mental health on the mental health of children exposed to political violence examined it as having a direct [14,33], mediating [14,32,34-37], or a moderating effect (buffering effect) [38]. Our previous study of the mental health of preschoolers in Gaza found that factors associated with resilience were maternal rated good health, higher maternal level of education, and less child exposure to traumatic events. Factors associated with vulnerability in child's mental health were poor maternal mental health, and male gender [28].

Because so far no published studies have addressed HRQOL of children in war, we used this measuring scheme to examine how violence and deprivation affect quality of life of preschoolers in the Gaza Strip, who have experienced longstanding military conflict throughout their lives. We used the PedsQL 4.0 to conceptualize the effects of war on children beyond PTSD. While there is no reference data for quality of life for children in war, we expect that the quality of life of Palestinian preschoolers is much worse than that of the American reference population, and similar to that of American children with chronic diseases. Based on review of factors associated with mental health of children exposed to war [14,17-20,25,30-32,38], we postulate that the health-related quality of life declines with both direct and indirect exposures to violence, and with increased number of exposures to traumatic events. Beit Hanoun, one of the districts we sampled from, lies in the borders and has high level of confrontation, so we expect that health-related quality of life of preschoolers in Beit Hanoun to be lower than the rest of the study sample. In addition to exposures to violence, we hypothesize that other factors are negatively associated with health-related quality of life and physical health and psychosocial health subscales: deprivation, low maternal education, inadequate social support, and male gender. In addition, we expected that HRQOL would correlate with other measures of health such as stunting and the child's mental health as well as with mental and self-reported overall health of the mother. As a secondary aim, we explored the feasibility, reliability and content validity of PedsQL in the study sample to document its relevance for the study of children exposed to political violence.

## Methods

### Description of target population and sampling design

As previously described [28], we conducted a cross-sectional kindergarten-based survey of children 3-6 years old in the Gaza Strip, the region most adversely affected by the political conflict and deprivation in Palestine. A list of 964 kindergartens in the Gaza Strip was provided by the Palestinian Ministry of Education. We stratified the list according to two criteria to ensure a broad representation of kindergartens across the Gaza Strip: 1) locality (defined as a permanently inhabited place with an independent municipal administration [12]); and 2) type of administration (public, private, and United Nations Relief and Works Agency for Palestine Refugees in the Near East [UNRWA]). In six of the eight localities, one kindergarten was randomly selected. Due to the larger population in the remaining two localities, two kindergartens were selected there. The random sample of kindergartens included four cities, two villages, and two camps. Six of the randomly selected kindergartens were from Gaza City, Der El Balah City, Rafah and Khan Younis Cities. Two were selected from Beach camp and Nusirate camp, and two from Beit Hanoun and Zwaida villages. These represent the different types of kindergartens found in Gaza: two are UNRWA, two are public, and six are private kindergartens. The Human Subjects Office, College of Letters and Sciences at the University of Wisconsin-Madison, approved the study protocol in December 2006.

### Recruitment

The field manager called the parents using phone numbers in registration books, briefed them about the study, and invited the mothers to come to the kindergartens to participate. Because a written consent form is not culturally relevant in the Gaza Strip, verbal informed consent was obtained from the mothers following a description of the study. The mothers were given the option to skip any questions they did not feel comfortable answering, and to temporarily or permanently stop the interview. The response rate was 100%, consistent with the high response rates in previous cross-sectional surveys and Palestinian Demographic Health Surveys (2000, 2002, 2004, and 2006), which ranged from 96-98% [20,39-42].

### Variables

#### Health-related quality of life assessment

HRQOL was measured using PedsQL 4.0- parent report [12]. We used two versions of PedsQL: one for toddlers (2-4 years) and one for children (5-7 years). We translated both versions to Arabic and then translated back to English following recommended guidelines [43]. The original English version of the PedsQL has been extensively validated, with Cronbach's alpha (a reliability measure of the intercorrelation of items) around 0.90 [7,12]. The reference population was a large (13,878 children) diverse sample of American children whose parents were recruited from general pediatric clinics, subspecialty clinics, and hospitals in which children were being seen for well-child checks, mild acute illness, or chronic illness care (26.8%), and from a State Children's Health Insurance Program (SCHIP) in California (73.2%), including mostly low income children [44]. The 23-item PedsQL 4.0 Generic Core Scales encompass: physical functioning (8 items); emotional functioning (5 items); social functioning (5 items); and school functioning (5 items). A 5-point Likert response scale is used for parent proxy-report (0 = never a problem; 1 = almost never a problem; 2 = sometimes a problem; 3 = often a problem; 4 = almost always a problem). Items are reverse-scored and linearly transformed to a 0-100 scale (0 = 100, 1 = 75, 2 = 50, 3 = 25, 4 = 0), so that higher scores indicate better HRQOL [45]. In our study, we defined "severely impaired" as a total score falling at least one standard deviation below the reference population mean [12]. For descriptive purposes we also report physical, emotional, social, and school functioning subscores. In addition, we computed a psychosocial health summary score as the sum of the items divided by the number of items answered in the emotional, social, and school functioning subscales.

#### Child and Household Measures

##### Child age

The age of the child as months completed was calculated from the date of birth as reported by the mother.

##### Nutritional status

We measured children's height to examine stunting (low height for age) in the study sample. We used Z-scores (obtained using "WHO ANTHRO 2005" for children up to five years old; because the WHO program does not cover children older than 5 years, we used "NutriSurvey for Windows" for six-year-olds) of less than 2.0 as the cut-off point for stunting [46]. We defined moderate nutrition as Z-scores between -2.0 and 0.0 and nutritional resilience as a Z-score zero or above.

##### Child mental health

Child mental health status was measured as the total difficulties score from the Strength and Difficulties Questionnaire (SDQ) [47]. SDQ measures behavior problems, emotional symptoms, hyperactivity/inattention, peer relationship problems, and pro-social behavior. Normal mental health is defined as total scores on SDQ ≤13, borderline cases as total scores between 13 and 17, and poor mental health as total scores on SDQ ≥17 [47].

##### Exposure to violence

Rating was based on adding child's exposure to traumatic events in the past year based on the 22-item Gaza Traumatic Event Checklist [20]. It was examined as both a summary measure and individual exposures. In the regression models, we either included the summary score or the individual exposures (but not combined). For descriptive analysis, we recoded this variable into 3 categories reflecting 3 levels of exposures based on the number of exposure to traumatic events; level 1 (1-4 exposures), level 2 (5-9 exposures), and level 3 (more than 9 exposures).

##### Confrontation level

Measured by asking the mothers if their dwelling lies in a direct military confrontation area, where fighting between Israeli soldiers and the Palestinians and military operations are frequent (Yes/No).

##### Deprivation

This rating was constructed by summing the responses (Yes/No) to the following dichotomous items: in the last three months, family did not have enough money for living expenses, did not have money to pay the bills, and the mother felt that her child was deprived [25,36]. It was then recoded into a binary variable, based on reporting one or more forms of deprivation (Yes/No).

##### Social support

The perceived social support factor was drawn from the items on the Social Provisions Scale [48]. Rating was based on the (Yes/No) answer to the following items: mother had someone to count on for help, had friends and family to make her happy and secure, had somebody she trusts to talk about problems, and had someone with whom she feels intimacy [36]. It was then recoded into a binary variable, having any one of these types of social support (Yes/No). In our sample, Cronbach's alpha for social support was 0.75.

##### Maternal mental health

Ratings were based on the General Health Questionnaire (GHQ-28). It covers severe depression and suicide risk, anxiety and insomnia, social dysfunction, and somatic symptoms [49]. GHQ-28 scores above the cutoff of 4/5 are considered to be possible psychiatric 'cases' [50]. In a previous study, Cronbach's alpha coefficients of reliability for the GHQ-28 was 0.91 and test-retest coefficient after six months was 0.90 [49]. A validation study of the GHQ-28, in comparison with the Clinical Interview Schedule, yielded a sensitivity and specificity to diagnose possible psychiatric cases which were 88.0% and 84.2%, respectively [49]. In our study, Cronbach's alpha of GHQ-28 was 0.87.

##### Maternal self-rated health

We measured health perceptions through the question, "At the present time, would you say your health is excellent, very good, good, fair, or poor?" We recoded this variable into a binary variable: fair or poor versus good/very good/excellent.

We translated the study questionnaire to Arabic and piloted it among mothers of 35 preschoolers in the Gaza Strip. Finally, we revised the questionnaire and administered it to the mothers in the children's kindergartens based on a defined protocol.

## Analysis

The dataset comprised 350 children and their mothers and was analyzed with the SAS statistical package [51]. We used mean values, standard deviations, and percentages to characterize the sample. Because of the special condition in the locality Beit Hanoun (the most disadvantaged among the eight examined localities in terms of violence and material deprivation), we also present separate analysis for this city.

We examined the feasibility of the PedsQL 4.0 as a population health measure among Palestinian children based on the percentage of missing values for each item in the scale, thus attesting whether mothers were able to provide quality data regarding their children's HRQOL [12]. We also examined the responsiveness of the PedsQL and the possible existence of floor and ceiling effects by determining the percentage of scores at the extremes of the scaling range (maximum and minimum) [7]. We examined the scale's internal consistency reliability by calculating Cronbach's alpha for PedsQL 4.0 parent report for both the toddler and child version.

We assessed PedsQL using effect size by examining the differences in PedsQL scores between our sample means and the means in the reference population. We determined the effect size by taking the differences between the two means divided by the standard deviation of the scores in the reference population. Effect sizes for differences in means are designated as small (0.20), medium (0.50), and large (≥0.80) in magnitude [44]. In addition to estimating the overall HRQOL score and domain subscales scores, we calculated the mean scores by locality and among the different groups. Grouping was based on child characteristics and household factors. We used multivariable regression analysis to examine child and household factors associated with HRQOL score, physical health, and psychosocial health. The final model included all variables with a p-value ≤ 0.2. Tests of significance were two-sided. The fit of linear regression models was examined via analysis of variance (ANOVA test). Four models were constructed for each HRQOL total scores and psychosocial health and physical health subscales. Model 1 included child age and gender; model 2 included age, gender, exposure to traumatic events (examined either as summary scale or as individual items); model 3 included age, gender, exposure to traumatic events, and household factors; and model 4 included age, gender, exposure to traumatic events, household factors, and maternal factors.

## Results

### Sample characteristics

Table 1 shows the study sample characteristics. Mean child age was 59 months (4.9 years) and about half (49%) were females. About 60% of the mothers had poor mental health. Table 2 shows that the most prevalent exposures were hearing sonic sounds of jetfighters, watching mutilated bodies on television, and hearing shelling of houses. Table 3 shows that more than half of the study participants had no money to pay bills; had no meat, fish, fruits, or vegetables most days of the week; and were on food assistance.

**Table 1:** Characteristics of 350 preschool children in Gaza, 2007 according to responses to a series of standardized questionnaires administered to their mothers

	% (unless otherwise indicated)	
	**Study sample (N = 350)**	**Beit Hanoun (N = 35)**

**Child factors**		

Age in months [mean (SD)]	59 (8%)	61 (7%)

Females	49	60

Low birth weight (< 2.5 Kg)	7	9

Adequate nutrition ^1^	37	29

Stunting ^2^	15	9

Number of exposures to violence [mean (SD)]	6 (3%)	12 (3%)

Children with normal mental health	40	14

Children with poor mental health	37	69

**Household factors**		

Maternal age [mean (SD)]	31 (6%)	31 (5%)

Maternal elementary schooling only Education	10	26

Maternal self-rated health as fair or poor	32	40

Poor maternal mental health 3	60	89

Reported any form of social support 4	38	63

Living in a direct military confrontation area	25	94

Family moved in the past 2 years	19	29

Reported any form of deprivation 5	23	54

Receiving food assistance on regular basis	51	91

Average household income in US dollars * [mean (SD)]	205 (174%)	136 (169%)

Family size [mean (SD)]	8 (4%)	10 (4%)

Number of persons in household above 18 years [mean (SD)]	3 (2%)	4 (3%)

Number of children < 18 years [mean (SD)]	5 (3%)	6 (2%)

Number of children < 5 years [mean (SD)]	2 (1%)	3 (1%)

^1 ^Adequate nutrition: Height-for-age Z-score ≥0

^2 ^Stunting: Height-for-age < 2 SD from the population mean

^3 ^Total score on General Health Questionnaire ≥5

^4^The mother had someone to trust, to count on for help, had someone with whom she feels intimacy, or had family and friends that make her happy and secure.

^5 ^Family did not have money for living expenses, did not have money to pay the bills, or mother felt her child is deprived

* The estimate was based on 120 households.

**Table 2:** Frequency of child exposure to traumatic events^1 ^in the Gaza Strip, 2007 based on mother report, (N = 350)

Experience	Study Sample (N = 350)%	Study sample excluding those from Beit Hanoun (N = 315)%	Beit Hanoun (N = 35)%	P-value
Heard sonic sounds of jetfighters	94	94	94	0.94

Watched mutilated bodies or injured people on TV	93	93	94	0.83

Heard shelling by artillery	84	83	100	**0.007**

Witnessed signs of shelling on ground	50	45	97	**< 0.001**

Witnessed bombardment at houses	42	36	97	**< 0.001**

Witnessed assignation of people by rockets	28	23	77	**< 0.001**

Deprived of food, water and electricity during incursion	25	19	86	**< 0.001**

Witnessed firing on houses	24	16	97	**< 0.001**

Detained in house during incursion	23	17	86	**< 0.001**

Heard of killing of a close relative	23	20	46	**0.001**

Heard of a killing of a friend	16	15	31	**0.01**

Witnessed of firing on your house	13	8	60	**< 0.001**

Prevented from using toilet and getting out of his room during incursion	12	7	54	**< 0.001**

Destruction and stealing of his personal things by soldiers	7	2	54	**< 0.001**

Exposed to firing in an attempt to scare him	6	4	26	**< 0.001**

Injured due to bombardment of his house	5	4	17	**< 0.001**

Was beaten during incursion	5	3	17	**< 0.001**

Threatened of killing one of family members during incursion	4	2	23	**< 0.001**

Used as a barrier in the process or arresting a neighbor	2	2	6	0.15

^1^: Based on the 22-item-Gaza Traumatic Event Checklist [20]

^2^: Significant differences between Beit Hanoun and the rest of the study sample based on one-way Anova

**Table 3:** Frequency of material and food deprivation in the Gaza Strip, 2007 three months prior the survey (N = 350)

	%
No meat/chicken/fish most days of week	56

No fruits and vegetables most days of week	61

Insufficient food most days of the week	42

On food assistance	51

Shortage of money for living expenses	12

No money to pay bills	18

Mother thought her child was deprived	16

Compared to the overall study sample, Beit Hanoun City was more disadvantaged in terms of exposures to violence, deprivation, the concentration of mothers and children with poor mental health (89% and 69%, respectively), and proportion of mothers with only elementary schooling. However, the prevalence of stunting in Beit Hanoun was less than that of the overall study sample (9% versus 15%, respectively).

### Feasibility, reliability, and correlates of PedsQL 4.0 in Palestinian children

In our study there were no missing item responses in any of the two examined versions, suggesting that the PedsQL 4.0 is feasible among Palestinian preschoolers and that mothers are able to provide quality data regarding their children's HRQOL [12]. Cronbach's alpha of PedsQL 4.0 among toddlers and children 5-7 years of age were 0.91 and 0.85, respectively, similar to those reported by Varni for the younger group, but slightly below in the older group [44].

In line with studies of validity and reliability of PedsQL 4.0 [19], we did not find floor effect in the total score nor in any domain subscale, as no scale had more than 0.6% of respondents scoring the minimum. Minimal ceiling effects existed for physical health, social functioning, and school functioning (from 2% to 13%).

PedsQL scores were related to the toddler's and child's SDQ scores in the expected direction (r = -0.32, -0.33, respectively), with a stronger relationship (r = -0.33, -0.42, respectively) for the psychosocial subscale. The PedsQL total score was non-significantly higher for stunted children (p = 0.20). The PedsQL total score was non-significantly higher with better maternal mental health and better self-reported health (p = 0.06, p = 0.67, respectively). Mothers with poor mental health reported lower scores in psychosocial health (p = 0.02) and emotional functioning (p = 0.003) in their children, while those who rated their health as fair or poor reported lower scores in emotional functioning in their children (p < 0.001).

### Mean scores in HRQOL, psychosocial, and physical health

Children from Beit Hanoun City had significantly lower scores in all PedsQL domains except for physical functioning and school functioning, than those from other localities (Table 4). The effect sizes between the mean scores among children from Beit Hanoun and the rest of the study sample ranged from 0.2 for physical health to 0.9 for emotional functioning.

**Table 4:** Mother-Report: Means and Standard Deviations for the PedsQL™ 4.0 Generic Core Scales among preschoolers in Gaza and Comparisons American reference population scores.

Scale	**Study Sample mean (N = 350)**^**a**^		**Beit Hanoun sample mean (N = 35)**^**b**^			
	**Mean**	**SD**	**Mean**	**SD**	**Differences**	**Beit Hanoun vs. Study sample**^**2**^

Total Score ^1^	62	16	54	17	b < a**	0.5

Physical Health	69	20	66	20	b < a	0.2

Psychosocial Health	58	16	48	18	b < a***	0.6

Emotional Functioning	50	16	35	16	b < a***	0.9

Social Functioning	63	24	54	28	b < a*	0.4

School Functioning	61	21	55	25	b < a	0.3

^1: ^Total score is from 0-100 with higher score indicating better HRQOL

^2^: Effect sizes calculated for differences in means between American reference population and Palestinian children are designated as small (0.20), medium (0.50), and large (≥0.80) in magnitude

*p < 0.05, **p < 0.01, ***p < 0.001 based on student's t-test

Preschoolers in Gaza had more impaired overall HRQOL than children in the American reference population, with large effect size (62 vs. 81, effect size 1.2) (Table 5). Compared to the reference population, the mothers reported severely impaired HRQOL, psychosocial health, and emotional functioning. Based on mother's ratings, the lowest subscale score was for emotional functioning (50) and highest was for physical functioning (69).

**Table 5:** Scale descriptive for PedsQL 4.0 Generic Core Scales for (350) preschoolers in the Gaza Strip, 2007

Scale	**Reference values**^**1**^		**Sample mean (N = 350)**^**2**^			
	**Mean**	**SD**	**Mean**	**SD**	**Differences**	**Sample vs. Reference Effect Size**^**3**^

Total Score ^2^	81	16	62	16	b < a*	1.2

Physical Health	83	20	69	20	b < a*	0.7

Psychosocial Health	80	16	58	16	b < a*	1.4

Emotional Functioning	80	17	50	16	b < a*	1.8

Social Functioning	82	20	63	24	b < a*	0.9

School Functioning	77	20	61	21	b < a*	0.8

^1^: Reference values provided by the author of PedsQL 4.0, based on American reference population [12]

^2^:Total score is from 0-100 with higher score indicating better HRQOL

^3^: Effect sizes for differences in means are designated as small (0.20), medium (0.50), and large (≥0.80) in magnitude

*p < 0.0001, based on student's t-test.

### Group differences in PedsQL scores (Tables 6 and 7)

**Table 6:** Mean Unadjusted PedsQL 4.0 Generic Core Scales Total Scale Score, Psychosocial and Physical Health for (350) preschoolers in the Gaza Strip, 2007

		PedsQL 4.0 Total Score	Difference	Psycho-social Health	Difference	Physical Health	Difference
	**N**	**Mean (SD)**		**Mean (SD)**		**Mean (SD)**	

**Demographics** Gender							

Female ^a^	170	63 (16)	b < a	59 (16)	b < a	71(20)	b < a

Male ^b^	180	61(15)		57 (16)		68(20)	

Age							

Toddler (3-4) ^a^	184	64 (16)	b < a	60 (17)	b < a	72(20)	b < a*

Young child (5-6) ^b^	166	60 (15)		56 (16)		67(19)	

**Correlates** Nutritional status							

Stunting ^a^	52	64 (14)	b < a	64 (14)	b < a*	71(18)	b < a***

Moderate ^b^	168	62 (16)	c < a	58 (17)	c < a*	62(16)	c < a***

Adequate ^c^	130	62 (16)		58 (16)		58(16)	c < b*

Child mental health^1^							

Poor ^a^	129	57 (15)	a < b**	52 (16)	a < b**	66(19)	a < b

Borderline ^b^	82	63(16)	a < c***	59 (15)	a < c***	71(21)	a < c

Normal ^c^	139	66 (15)	b < c	63 (15)	b < c	72(19)	b < c

Maternal mental health^2^							

Poor ^a^	209	61(16)	a < b	57 (17)	a < b	66(19)	a < b

Normal ^b^	141	64(15)		61(15)		71(20)	

Maternal self-rated health							

Fair or poor ^a^	112	61 (16)	a < b	57 (16)	a < b	70(20)	b < a

Good/very good/excellent ^b^	238	63(16)		59 (16)		69(20)	

Level of Exposure to traumatic events							

Level 1 : (1-4 traumatic events) ^a^	158	65 (16)	b < a*	62(16)	b < a**	72(20)	

Level 2: (5-9 traumatic events) ^b^	145	61(14)	c < a***	57(14)	c < a***	68(20)	

Level 3: (10-19 traumatic events) ^c^	47	55(16)	c < b*	50 (18)	c < b**	67(18)	

Maternal education							

Elementary ^a^	34	55(14)	a < b**	52 (15)	a < b*	61(19)	a < b

Above elementary ^b^	316	63(16)		59 (16)		70(20)	

Living in direct military confrontation area							

Yes ^a^	88	58(15)	a < b**	52 (16)	a < b***	69(17)	a < b

No ^b^	262	64 (16)		61(16)		70(20)	

Moved in the past 2 years							

Yes ^a^	65	62 (16)		58 (17)		69(20)	a < b

No ^b^	285	62(16)		58 (16)		70(20)	

Deprivation							

Yes ^a^	83	59(15)	a < b	54 (16)	a < b**	69(18)	a < b

No ^b^	267	63(16)		60 (16)		70(20)	

Social support							

No ^a^	218	62(15)		59 (16)	b < a	69(19)	a < b

Yes ^b^	132	62(16)		57 (17)		70(20)	

**Total score**	**350**	**62(16)**		**58 (16)**		**69(20)**	

1: Child mental health was measured using Strength and Difficulties Questionnaire (SDQ)

2: Maternal mental health is measured using General Health Questionnaire (GHQ)

*p < 0.05, **p < 0.01, ***p < 0.001 based on student's t-test

**Table 7:** Mean Unadjusted PedsQL 4.0 Generic Core Scales Emotional, Social, and School Functioning, Gaza, 2007

		Emotional functioning	Difference	Social functioning	Difference	School functioning	Difference
	**N**	**Mean (SD)**		**Mean (SD)**		**Mean (SD)**	

**Demographics**							

Gender							

Female^a^	170	51(17)	b < a	65(24)	b < a	62(22)	b < a

Male^b^	180	50(16)		62(24)		61(20)	

Age							

Toddler (3-4)^a^	184	51(16)	b < a	66(24)	b < a*	63(22)	b < a

Young child (5-6)^b^	166	49(16)		60(23)		59(19)	

**Correlates**							

Nutritional status							

Stunting^a^	52	51(14)	b < a	68(24)	b < a	63(19)	b < a

Moderate^b^	168	49(17)	b < c	63(23)	c < b	61(21)(22)	c < b

Adequate^c^	130	51(16)		62(24)	c < a	60(22)	c < a

Child mental health^1^							

Poor ^a^	129	43(16)	a < b***	57(25)	a < b*	58(21)	a < b

Borderline ^b^	82	53(15)	a < c***	65(22)	a < c***	59922)	a < c***

Normal ^c^	139	55(14)	b < c	69(22)	b < c	66(19)	b < c*

Level of Exposure to traumatic events							

Level 1 : (1-4 traumatic events) ^a^	158	55(15)	b < a***	67(24)	b < a	64(20)	b < a*

Level 2: (5-9 traumatic events) ^b^	145	49(15)	c < a***	64(22)	c < a***	59(20)	c < a*

Level 3: (10-19 traumatic events) ^c^	47	38(17)	c < b***	52(25)	c < b**	59(24)	

Maternal mental health^2^							

Poor ^a^	209	48(18)	a < b**	62(25)	a < b	60(21)	a < b

Normal ^b^	141	53(14)		65(22)		63(20)	

Maternal self-rated health							

Fair or poor ^a^	112	45(17)	a < b***	65(23)	b < a	60(21)	a < b

Good/very good/or excellent^b^	238	53(15)		63(24)		62(21)	

Maternal education							

Elementary ^a^	34	44(17)	a < b*	55(25)	a < b*	59(18)	a < b

Above elementary ^b^	316	51(16)		64(24)		61(21)	

Living in direct military confrontation area							

Yes ^a^	88	42(17)	a < b***	56(23)	a < b***	57(22)	a < b*

No ^b^	262	53(15)		66(23)		63(20)	

Moved in the past 2 years							

Yes ^a^	65	51(17)	b < a	64(23)	b < a	59(21)	a < b

No ^b^	285	50(16)		63(24)		62(21)	

Deprivation							

Yes ^a^	83	43(18)	a < b***	61(23)	a < b	58(23)	a < b

No ^b^	267	52(15)		64(24)		62(20)	

Social support							

No ^a^	218	52(15)	b < a***	63(23)	a < b	62(20)	b < a

Yes ^b^	132	47(18)		65(24)		60(22)	

**Total Score**	**350**	**50(16)**		**63(24)**		**61(21)**	

1: Child mental health was measured using Strength and Difficulties Questionnaire (SDQ)

2: Maternal mental health is measured using General Health Questionnaire (GHQ)

*p < 0.05, **p < 0.01, ***p < 0.001 based on student's t-test

#### Child factors

Older children had lower (worse) physical health (67 vs. 72, p < 0.05), and social functioning scores (60 vs. 66, p < 0.05). Children with high levels of exposure to traumatic events had lower scores in HRQOL (55 vs. 65, p < 0.001), psychosocial health (50 vs. 62, p < 0.001), emotional (38 vs. 55, p < 0.001), social functioning (52 vs. 67, p < 0.001), and school functioning (59 vs. 64, p < 0.05).

#### Household factors

Children living in direct military confrontation areas had lower HRQOL (58 vs. 64, p < 0.01), psychosocial health (52 vs. 61, p < 0.001), emotional (42 vs. 53, p < 0.001), social (56 vs. 66, p < 0.001), and school functioning (57 vs. 63, p < 0.05). Children whose mothers reported at least one form of deprivation had lower scores in psychosocial functioning (54 vs. 60, p < 0.01), mainly emotional functioning (43 vs. 52, p < 0.001). Those whose mothers reported any form of social support had lower emotional functioning score (47 vs. 52, p < 0.001). Mothers with only elementary schooling reported lower scores in HRQOL (55 vs. 63, p < 0.01), and lower psychosocial health (52 vs. 59, p < 0.05).

### Multivariable model for HRQOL, physical health, and psychosocial health

Tables 8 and 9 show factors associated with HRQOL, psychosocial health, and physical health in a multivariable regression analysis. Factors associated with lower HRQOL were child older age (p = 0.01), male gender (p = 0.05), and more exposures to traumatic events (p < 0.001). Factors associated with lower psychosocial health were child older age (p = 0.03), history of deprivation of food, water, and electricity during incursion (p < 0.001), and witnessing assassination of people by rockets (p = 0.05). Factors associated with lower physical health were child older age (p = 0.01), child being deprived of food, water, and electricity during incursion (p < 0.001), and heard of a killing of a friend (p = 0.03). Child gender was not associated with either psychosocial or physical health.

**Table 8:** Factors associated with PedsQL 4.0 Generic Core Scales Scores among (350) preschoolers in the Gaza Strip, 2007

	Health-related quality of life total score		
	**Beta**	**SE Beta**	**p-value**

Child age (month)^1^	-0.26	0.10	0.01

Child gender (male)^2^	-3.16	1.61	0.05

Number of exposure to traumatic events^3^	-1.31	0.25	< 0.001

Note: Beta: regression coefficient: difference in PedsQL score per unit difference in independent variable;

SE Beta: Standard Error of regression coefficient

^1^: For each additional month of age, child's score drops (worsens) by. 26 points

^2^: Girls have average score in PedsQL 3.16 more than boys

^3^: For each additional traumatic event, score drops by 1.31 points

**Table 9:** Factors associated with Psychosocial and Physical Health Scores among preschoolers in Gaza, 2007

	Psychosocial health			Physical health		
	**Beta**	**SE Beta**	**p-value**	**Beta**	**SE Beta**	**p-value**

Child age (month)	-0.22^1^	0.10	0.03	-0.32^2^	0.12	0.01

Being deprived of food, water, and electricity during incursion (Yes/No)	-8.25^3^	1.96	0.00	-8.10^4^	2.36	< 0.001

Witnessed assassination of people by rockets (Yes/No)	-3.70^5^	1.90	0.05			

Heard of a killing of a friend (Yes/No)				-5.98^6^	2.36	0.03

Note: Beta: regression coefficient: difference in PedsQL score per unit difference in independent variable;

SE Beta: Standard Error of regression coefficient

^1: ^For each additional month of age, child's score in psychosocial health drops (worsens) by .22 points

^2:^: For each additional month of age, child's score in Physical health drops (worsens) by .32 points

^3: ^Average score in psychosocial health among children deprived from food, water and electricity during incursion is 8.25 lower than those who were not deprived.

^4^: Average score in physical health among children deprived from food, water and electricity during incursion is 8.1 lower than those who were not deprived.

^5 ^: Those who witnessed assassination of people by rockets had average score in psychosocial health 3.7 lower than those who did not witness assassination of people

^6^: Those who heard killing of a friend had average score in physical health 5.98 lower than those who did not heard killing of a friend

## Discussion

Almost 65% of the Palestinian mothers in the Gaza Strip reported severely impaired HRQOL, psychosocial, and emotional functioning in their children. Compared to US children, the average total score of 62 was comparable to that of children with severe cardiac diseases (76), with end-stage renal disease (ESRD) (69), children receiving chemotherapy and radiation for the treatment of newly-diagnosed cancer (69), children with cerebral palsy (69) [45], and children with Attention-Deficit/Hyperactivity Disorder (AD/HD) (70) [8]. In the absence of comparable norms in Arabic children, the comparisons between children exposed to violence and deprivation and US children with chronic diseases are helpful to understand the possible magnitude of impact of war on HRQOL [8]. This approach, however, has some obvious limitations, namely, the inability to differentiate whether the very low HRQOL compared to the US reference is due to the direct effects of violence or to living in a resource-poor country or setting. The use of the PedsQL internationally has grown in recent years, and provides some additional insight. In a study among children from low income sectors in Argentina, the mean score in proxy reported PedsQL was 80 among children age 2-4 and 74 for age 5-7 [10]. In another study in Iran, the mean score was 73 for children mostly age 8-12 [11]. Although these scores are lower than the 80-88 in the US reference population, they are considerably higher than the 62 we found in the Palestinian sample, indicating the potential additional impact of violence. In fact, the mean score in Palestine is comparable to that of children with attention deficit in Iran of 57.

Compared to the overall study sample, Beit Hanoun City was more disadvantaged in terms of violence and deprivation. Beit Hanoun is in northern Gaza and is only 6 km away from the Israeli town of Sderot, and a few hundred meters from the Israeli-Palestinian border and has been described by an UNRWA representative as "a real humanitarian disaster" [29]. Based on our study, children from Beit Hanoun had the lowest scores in all PedsQL scales. Mental health was particularly affected, with 68.6% of Beit Hanoun children having poor mental health. As expected and consistent with previous research [10], physical health was least affected by deprivation and violence. Surprisingly, fewer children in Beit Hanoun showed stunting compared to the overall study sample. This may be explained by the higher percentage of the households in Beit Hanoun receiving food assistance on a regular basis compared to the overall study sample at the time of our study (91% versus 51%, respectively).

Older children in our sample had worse HRQOL according to their mothers. This is in alignment with a study of more than 5,000 preschoolers in the US which found that parents of older children tended to view them as having slightly lower health-related quality of life [31]. This may be explained by more exposures to traumatic events among older children which might have a cumulative effect, and therefore more psychosocial symptoms, compared to younger children [52].

Except for poor child mental health and living in a direct military confrontation area, each form of adversity has different associations with PedsQL. Children's HRQOL was associated with both violent and nonviolent exposure to traumatic events. In line with previous studies among preschool children in Gaza and among displaced Israeli children after Iraqi Scud missile attacks, exposures to traumatic events were associated with poor psychosocial health [20,53]. Based on multivariate regression analysis, social support was not associated with HRQOL nor physical and psychosocial health scores in our study. The latter result is consistent with a previous study of factors associated with behavioral and cognitive resilience in England [35], but not with a number of studies that found that social network is among the protective factors that are likely to alleviate negative effects of war [54]. This may be explained by the high prevalence of poor mental health among the mothers (60% based on our study sample) and thereby the inability to offer support and help to buffer the negative effects of war.

None of the maternal factors were associated with HRQOL in children, which is inconsistent with previous studies in Gaza [55], among children in Bosnia and Herzegovina [31], among Israeli children after a Scud Missile attack [56], and also inconsistent with our previous study showing that poor maternal mental health was associated with poor child mental health [30].

Although this study adds to the trauma literature, it has several limitations. First, this is an observational cross-sectional study so we cannot examine causality. The study provides a snapshot of children's HRQOL during political violence and ongoing war, and cannot address issues related to children's pre-war status. Furthermore, parental report can be affected by child age and mother's self-rated health [10]. The biased mother may report both higher child exposure to traumatic events and lower HRQOL. However, the SCHIP comparison population (73% of the reference population) is actually already a lower standard than a completely stress-free population; they are lower-income families not eligible for Medicaid [45]. Finally, although the Arabic version of PedsQL which we developed seems feasible, reliable, and has content validity (it is correlated in the expected direction with other health measures), this is the first time this instrument was used in this population, and further evaluation of validity is needed.

This study is unique in several aspects. First, it is the first study we are aware of which highlights the health-related quality of life of preschool children exposed to wartime trauma, and offers insight into the causes of lower HRQOL in this population. Second, it is the first to examine how child nutrition and mental health relates to quality of life in these children. Third, the present study extends the small amount of literature on the effects of war on child health, and the relationship between maternal mental health, self-rated health, perceived social support, and education on children's HRQOL. Fourth, the study context provided a unique opportunity to examine the impact of both chronic and acute exposure to political violence on children's health.

## Conclusions

The PedsQL 4.0 scores evaluating children's quality of life were very low among Palestinian children exposed to political violence. Both violent and nonviolent exposures were associated with lower quality of life in children. These findings suggest there may be need for comprehensive interventions to support children exposed to political violence and deprivation, with the aim of improving their physical and psychosocial health to improve their health-related quality of life. Special attention should be given to Beit Hanoun City and other areas close to the border, as children in Beit Hanoun had the lowest scores on all PedsQL scales compared to those from other localities. The study findings warrant further research on children from other countries exposed to violence or deprivation to validate instrumentation and provide more comparisons. In the end, of course, the public health imperative would be to remove exposures to the stresses of war and violence [57].

Our findings have public health and clinical implications because they highlight the need for immediate psychosocial interventions targeting child health. Children requiring interventions can be identified through schools and primary health clinics, such as those run by the World Health Organization [58]. Among the interventions that seemed to be promising are cultural and recreational activities such as traditional dancing, art work, sports, drama, and puppetry. The involvement of teachers, parents, and other caregivers is vital in such programs so that they can support the well-being of children exposed to political violence and deprivation. Such interventions may consist of semi-structured group discussion meetings for mothers to support them and increase their sense of well-being, self-confidence, and ability to care for their children in this difficult situation, and to be their children's best healer [31].

Furthermore, interventions should take into consideration children's development of child psychiatric disorders, instead of only targeting PTSD. In addition to assessment and treatment, specialist practitioners (psychologists and psychiatrists) have an important role to play in setting up training for non-specialist staff, to enable them to detect and manage simple cases and refer the more complex ones to the sparse specialist resources in that area [58].

In conclusion, health and welfare policies, international organizations, and governments can make a substantial contribution to children's mental health and wellbeing, even under the most adverse circumstances [58]. International organizations such as the United Nations and UNICEF can play a major role in providing support through socioeconomic stability, education, alternative coping strategies, and awareness of the impact of trauma [58].

Finally, special attention should be given to those living near the border in zones of conflict, where there is constant threat and ongoing trauma and where the majority of children with poor health-related quality of life found in this study are concentrated. Although there is some research evidence on strategies to prevent or minimize children's response to trauma through ongoing political conflict, these are more likely to be successful through agencies operating in the area, and in collaboration with schools, which are the main source of stability and safety for the children.

## List of Abbreviations

PTSD: Posttraumatic stress disorder; HRQOL: health-related quality of life; PedsQL: Pediatric Quality of Life Inventory parent report; UNRWA: United Nations Relief and Works Agency for Palestine Refugees in the Near East; GHQ: General Health Questionnaire; SDQ: Strength and Difficulties Questionnaire.

## Competing interests

The authors declare that they have no competing interests.

## Authors' contributions

SGM made contribution to the study design, data collection, statistical analysis, interpretation of data and the drafting of the manuscript. FJN contributed to the study design, statistical analysis, interpretation of data and drafting of the manuscript. MP guided the statistical analysis and contributed to the interpretation of results and to writing and editing the manuscript. MS contributed to study design, data collection, interpretation of data, and manuscript writing and editing. RC contributed to study design, interpretation of data, and manuscript writing and editing. AT contributed to the study design and data collection. All authors read and approved the final manuscript.

## Pre-publication history

The pre-publication history for this paper can be accessed here:

http://www.biomedcentral.com/1471-2458/11/253/prepub
